# Automatic detection of tweets reporting cases of influenza like illnesses in Australia

**DOI:** 10.1186/2047-2501-3-S1-S4

**Published:** 2015-02-24

**Authors:** Guido Zuccon, Sankalp Khanna, Anthony Nguyen, Justin Boyle, Matthew Hamlet, Mark Cameron

**Affiliations:** 1 Information Systems School, Queensland University of Technology, Brisbane, Australia; 2 The Australian e-Health Research Centre, CSIRO Digital Productivity Flagship, Brisbane, Australia; 3 CSIRO Digital Productivity Flagship, Canberra, Australia

## Abstract

Early detection of disease outbreaks is critical for disease spread control and management. In this work we investigate the suitability of statistical machine learning approaches to automatically detect Twitter messages (tweets) that are likely to report cases of possible influenza like illnesses (ILI). Empirical results obtained on a large set of tweets originating from the state of Victoria, Australia, in a 3.5 month period show evidence that machine learning classifiers are effective in identifying tweets that mention possible cases of ILI (up to 0.736 F-measure, i.e. the harmonic mean of precision and recall), regardless of the specific technique implemented by the classifier investigated in the study.

## Introduction

Early detection of disease outbreaks is a crucial capability for hospitals and public health officials to effectively allocate resources to control the spread of diseases and treat affected patients [1,2]. In Australia, state agencies keep track of the number of patients tested positive for influenza and influenza like illnesses (ILI). National initiatives attempt to obtain timely reporting through measures such as the Australian Sentinel Practices Research Network (ASPREN) (http://www.aspren.com.au/, last visited October 13, 2014), the National Health Call Centre Network (http://www.health.gov.au/internet/main/publishing.nsf/Content/national-health-call-centre-network-team-overview, last visited October 13, 2014) and community level surveillance through FluTracking (http://www.flutracking.net/, last visited October 13, 2014). These systems, although enlarging the population base that is monitored, suffer poor participation rates [3] and high costs.

**Figure 1:**
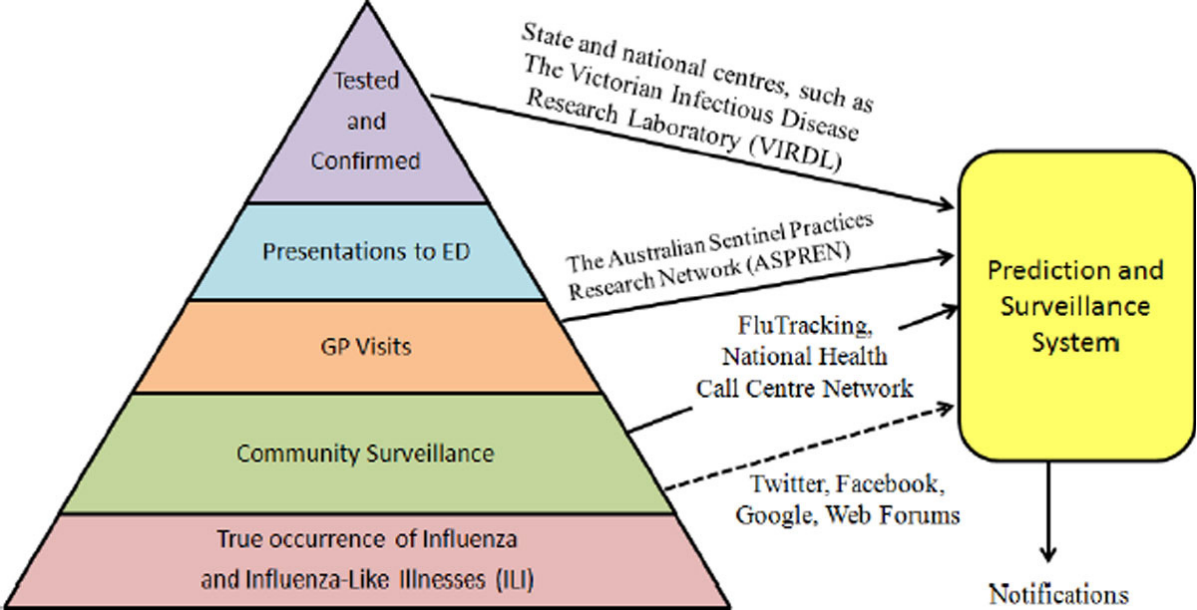
**Disease prevalence pyramid and notification data sources; dotted line is proposed**. Adapted from [10].

Figure 1 outlines the disease prevalence pyramid, where the width of each layer represents the population size involved or monitored. The benefits of expanding the data sources used to produce disease outbreak notifications to web data and social media [4], in particular Twitter, are numerous. The data is publicly available, its access cost is low, the participation rate is high (http://www.nielsen.com/us/en/insights/news/2010/australia-getting-more-social-online-as-facebook-leads-and-twitter-grows.html, last visited October 13, 2014) and the user base is generally broad, although not uniform with respect to age groups and geographic areas. By leveraging information published in Twitter, real time reporting across a large fraction of the population may be possible.

Previous studies that monitored Twitter in the US [5] and UK [6,7] have found that it is possible to produce highly correlated predictions for influenza-affected patients from the use of Twitter alone. However, the use of Twitter is not without its problems. The volume of tweets is exceedingly large, with users producing over 200 million tweets per day globally as of mid 2011 (http://blog.twitter.com/2011/06/200-million-tweets-per-day.html, last visited October 13, 2014). The content of a message is highly condensed and often expressed differently than natural language due to the size limitation of a tweet (140 characters). To render the data useful for predictions, it must be collected and analysed in real time, and its manual processing may not be timely nor cost efficient.

This necessitates an automatic system that can classify tweets reporting influenza cases with high accuracy. Previous work by Collier and Doan [8] has shown evidence that Naive Bayes (NB) and Support Vector Machine (SVM) classifiers, informed by a limited set of textual features created by extracting only terms contained in an health ontology, were able to classify tweets with respect to common syndromic categories.

This paper presents a study of detecting mentions of influenza from Twitter messages originating from Victoria, Australia, which is characterised by a smaller and more geographically diverse population (higher population density and high-speed/large-bandwidth internet access in metropolitan areas, and low population density and low-speed/small-bandwidth internet access in rural and regional areas) than those studied in previous work. The paper reports a thorough evaluation of an array of machine learning approaches for identifying Twitter messages that may indicate cases of ILI. Correlation with confirmed influenza cases was not within the scope of this work. Investigated methods go beyond the two popular classifiers tested previously by others, i.e. Naive Bayes and Support Vector Machine, expanding the analysis to other learning approaches such as decision trees (C4.5/J4.8, Random Forests, Logistic Model Trees), and perceptrons and regression models (Voted Perceptron, Linear Logistic regression, Multinomial Logistic Regression). The results suggest that machine learning techniques are able to discriminate among tweets containing mentions of influenza or relevant symptoms and irrelevant messages. In addition, our experiments show that SVM classifiers do not always return the highest performance, and alternative approaches (e.g. Multinomial Logistic Regression and Random Forests) return higher performance under specific settings. However, the results also reveal that there is only limited differences in performance across the different types of classifiers. This suggests that future research efforts on the detection of influenza related tweets should be directed beyond improving machine learning techniques, in particular addressing how disease outbreak monitoring systems should cope with false positive notifications produced by the proposed automatic methods, as well as true influenza mentions that are not captured (i.e. false negatives).

## Collection of Twitter messages and manual assessment

We obtained tweets posted in a 3.5 month period (May to August 2011), corresponding to the peak Australian flu season, all of which originated from users based in Victoria. This amounted to just over 13.5 million tweets. The tweets were captured using the ESA-AWTM architecture [9] that leverages the Twitter API, incorporating other services such as Yahoo! and Google maps to add extra metadata (e.g. location data). Initial analysis of these tweets revealed that around 0.1-0.2% of all 13.5 million tweets reported influenza cases [10]. In order to retain a significant amount of positive influenza reporting tweets from our data to train a classifier, but still be able to efficiently deploy computational methods, the Twitter stream was filtered to store only messages that contained keywords (and their derivatives) that may indicate cases of influenza. These keywords are listed in Table 1 and were selected by considering typical influenza symptoms as well as extending the keywords reported in previous research (e.g. Sadilek et al. [11] and Signorini et al. [12]). Note that re-tweets were removed. The application of this filtering process retained approximately 100,000 messages that were potentially influenza-related (0.75% of the initial data).

**Table 1:** Keywords that may indicate or exclude cases of influenza.

(a) Included keywords.						
flu	sick	headache	fever	unwell	chills	antibiotics

ache	cough	throat	cold	doctor	fatigued	tissues

stomach	runny	sneeze	pneumonia	down with	vomit	snot

influenza	Stuffy	tylenol	diarrhea	nausea	vicks	shivering

(b) Excluded keywords.						

		doctor who	jab	vaccine		

		shot	pandemic	fully sick		

		weather	Bieber	sick of		

**Figure 2:**
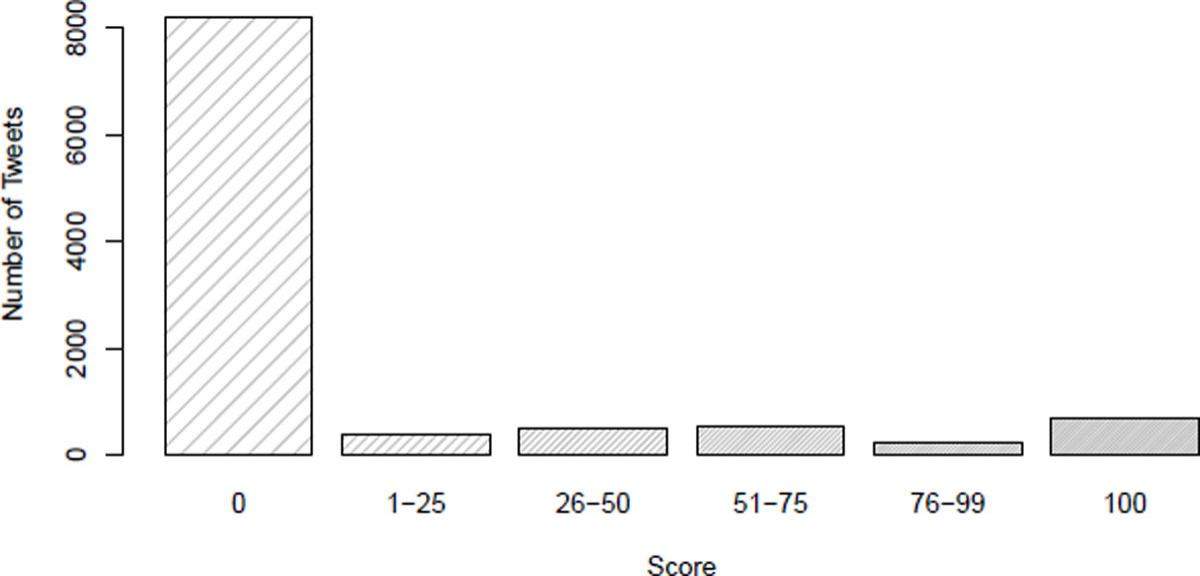
**Manually classified tweets, bucketed by likelihood of influenza or ILI score**.

From these approximately 100,000 tweets, a set of 10,483 tweets was randomly selected for manual classification to assess their likelihood of reporting a case of influenza. Seventeen volunteer assessors from The Australian e-Health Research Centre, CSIRO were asked to use a scale of 0-100 to select how likely they thought a tweet was representative of the user reporting a case of influenza (either with themselves or in others): 0 being no flu, 100 being certain of a flu. Figure 2 presents the results of this manual classification. The majority of filtered tweets (78.12%) were assessed as not related to ILI, although interestingly, 6.49% of tweets were assessed as certainly related to ILI.

In addition, 363 tweets, from the set of those that were manually classified, were assessed by multiple volunteers (three classifications per tweet on average), as an effort to measure inter-assessor agreement. The average standard deviation between the scores of tweets with multiple assessors was 4.89, indicating that the classification labels assigned by different assessors were comparable. Shorter tweets did have a higher standard deviation on average, as might be expected given that they contain less information. However, the differences between their scores were not judged large enough to require them to be treated differently. If a tweet was reviewed by more than one assessor, its average score was used for the remainder of the analysis.

## Automatic flu classification: statistical machine learning classifiers

### Problem definition

The problem of detecting ILI-related Twitter messages is casted into a binary classification problem: classify a tweet as being ILI-related or not. The collected ground truth indicates the likelihood of a tweet to be ILIrelated as a percentile score (i.e. between 0 and 100). Percentile scores were transformed into binary classes according to a threshold *th *which "defines an influenza related tweet". We refer to "definition of influenza related tweet" as the process of collapsing percentile scores assigned to tweets into a binary classification (i.e. ILI-related or not). Thus a "loose definition" corresponds to considering as influenza-related also tweets that have been assigned a relatively low score (e.g. 50). A "strict definition" instead corresponds to considering as influenza-related only tweets assigned with a high score (e.g. 100).

### Classifiers

The machine learning classification methods evaluated in this study for the task of identifying influenzarelated tweets are listed below. We investigated classifiers from three wide families of machine learning approaches, namely linear classifiers, support vector machines and decision trees. The corresponding Weka version 3.6.7 [13] implementations of these classifiers were used in the empirical experiments.

#### Linear classifiers

• standard Naive Bayes classifier.

• Linear logistic regression classifier (SimpleLogistic in Weka).

• multinomial logistic regression classifier with ridge estimator [14] (Logistic in Weka).

• Voted Perceptron which takes advantage of data that is linearly separable with large margins.

#### Support Vector Machine (SVM) classifiers

• Support Vector Machine (SMO in Weka) that uses a polynomial kernel and the sequential minimal optimization algorithm by Platt [15].

• Support Vector Machine (SPegasos in Weka) that uses a linear kernel and a stochastic variant of the primal estimated sub-gradient solver method by Shalev-Shwartz et al. [16].

#### Decision trees

• C4.5 Decision Tree learner (J48 in Weka) that builds a decision tree based on information entropy as measured on training data.

• Random Forest, an ensemble classifier method that constructs multiple decision trees during the training phase.

• Logistic model trees classifier (LMT in weka) where logistic regression functions are used at the leaves of the tree.

Details of the classification approaches and their implementation can be found in the Weka documentation; standard settings were used as defined in the software package.

### Features

The Medtex text analysis software [17] was used to extract features from the free-text of Twitter messages. Medtex is a medical text analysis platform that has been used in previous studies on cancer reporting [18-20], radiology reconciliation [21], and medical information retrieval [22]. Medtex architecture is characterised by a messaging framework built on the concept of message queues, producers (senders), and consumers (receivers). Because multiple message consumers can be set up in parallel to receive messages from the same queue, Medtex provides high text analysis throughput, making it an ideal framework for analysing large streams of Twitter data. While some of the specific clinical text analytic capabilities of Medtex were not used in this study (e.g. SNOMED CT and UMLS concept extraction), we extended the platform to include information extraction capabilities for specific entities that are present in Twitter messages, such as the presence of Twitter usernames (e.g. @Username), hash-tags indicating specific topics (e.g. #Topic), and emoticons (e.g. :-) and ;().

The features extracted from tweets using the Medtex software include:

• word tokens: strings identified by word boundaries such as white spaces and punctuation;

• word stems: the root stems of the word tokens (if available); stems were extracted using the Porter stemmer algorithm [23];

• word token n-grams: a continuous sequence of *n *word tokens in a tweet; we extracted both bi-grams and tri-grams (*n *= 2 and *n *= 3);

• binary feature representing the presence of a http:// token, identifying that the tweet contains a link to a web page;

• binary feature representing the presence of the token @ followed by a sequence of characters, identifying that the tweet has been directed to a Twitter user or presents a mention of that user;

• binary feature representing the presence of hashtags, i.e. tokens that start with the symbol # used to mark keywords or topics in a tweet;

• binary feature that represents the presence of a positive (negative) emoticon, i.e. a metacommunicative pictorial representation of a facial expression that conveys a positive emotion like happy, love, etc. (a list of positive and negative emoticons is given in Table 2);

**Table 2:** List of emoticons associated with positive and negative emotions.

	**Emoticons**
Positive Emotions	:) :=) :-) :D :=D :-D :d :=d :-d 8) 8=) 8-) B) B=) B-) :o :=o :-o :O :=O :-O :* :=* :-* :P :=P :-P :p :=p :-p :$ :-$ :=$ :\"> ]:) >:) 8-| B-| 8| B| 8=| B=| :x :-x :X :-X :# :-# :=x :=X :=# :? :-? :=?
Negative Emotions	:( :=( :-( ;( ;-( ;=( (:| :| :=| :-| :^) |-) I-) I=) |( |-( |=( |-() :& :-& :=& :@ :-@ :=@ x( x-( x=( X( X-( X=( :S :-S :=S :s :-s :=s

A total of 26,698 unique features formed the feature vocabulary for the entire set of annotated tweets used in the experiments reported in this article.

## Experimental settings

To evaluate the effectiveness of the machine learning approaches investigated in this article, we set up an evaluation framework that consisted of a first set of experiments using the 10-fold cross-validation methodology and a subsequent set of experiments where the classification models learnt in the cross-fold experiments were validated on unseen data (i.e. data not used for creating the models).

To this aim, we first constructed a balanced dataset for cross-validation experiments, that contained an equal number of positive (influenza-related) and negative (not influenza-related) instances. Specifically, the dataset contained 90% of the positive instances (i.e. tweets that had been annotated as being influenzarelated) and an equal amount of negative instances. These instances were randomly sampled from the respective classes. This dataset was subsequently randomly partitioned in 10 folds, and for each iteration of the cross-validation algorithm a unique combination of 9 folds were used for learning a classification model and the excluded one was used for testing the obtained model.

A second dataset was then formed by combining the remaining 10% of positive instances with the remaining amount of negative instances: this dataset was used to validate on unseen data the models learnt through cross-validation.

The described procedure was iterated for each 'likelihood of influenza score' threshold level (*th*), i.e. datasets were constructed for each threshold value: datasets varied in size across threshold values, due to the difference in number of positive instances when considering strict (e.g. *th *= 99) or relaxed (e.g. *th *= 49) thresholds for defining an influenza-related tweet. The use of unseen data to validate the models created using n-fold cross validation further reduces risks that the obtained results are due to over-fitting.

## Classification effectiveness

### Effectiveness on 10-fold cross validation

**Figure 3:**
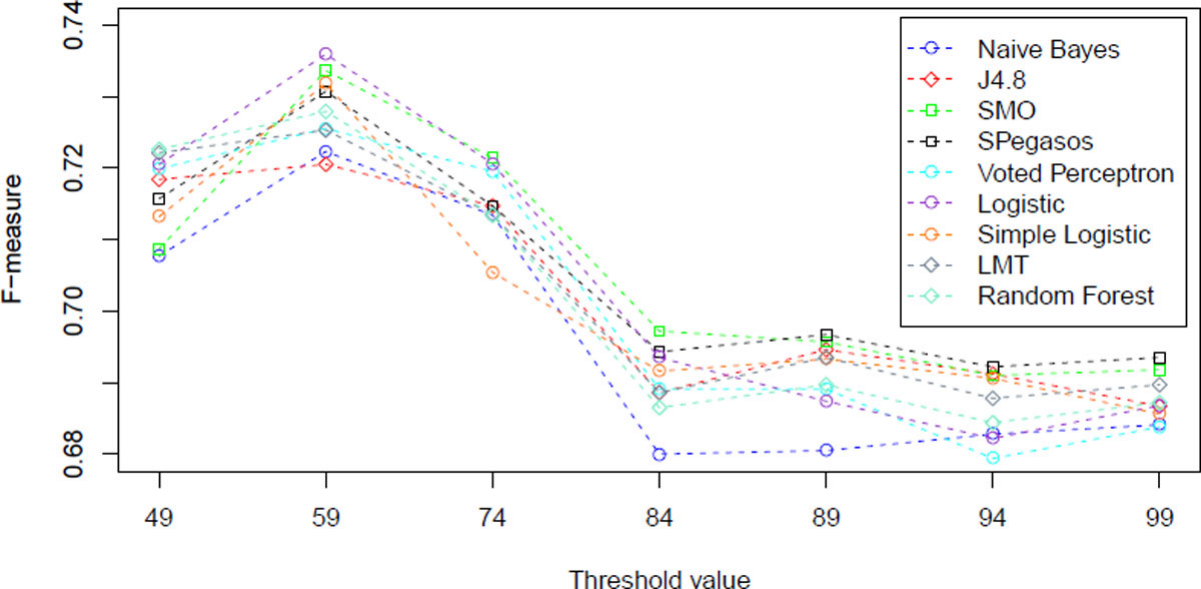
**Classifier effectiveness (F-measure) at different threshold values**.

Precision and recall values obtained by the studied classifiers in the 10-fold cross-validation experiments and with different threshold values *th *are detailed in Table 3 and the F-measure values are plotted in Figure 3. The F-measure summarises the precision-recall evaluation, being a balanced average of the two measures. Because the dataset used for the cross-validation experiments is balanced (same number of positive and negative instances), the two target classes (i.e. influenza and not-influenza) have equal importance. A majority class classifier then would achieve a maximum of 0.5 precision/recall/F-measure value. The confusion matrices for each setting of classifier and threshold value are reported in Table 4.

**Table 3:** Precision (prec) and recall (rec) values with respect to the 'likelihood of influenza' threshold level obtained by the studied classifiers when evaluated using 10-fold cross-validation.

	*th *> 49		*th *> 59		*th *> 74		*th *> 84		*th *> 89		*th *> 94		*th *> 99	
	rec	prec	rec	prec	rec	prec	rec	prec	rec	prec	rec	prec	rec	prec
NaiveBayes	.752	.668	.752	.695	.742	.687	.736	.632	.732	.636	.734	.639	.734	.641
J4.8	.760	.682	.750	.693	.747	.685	.748	.638	**.755**	.643	.751	.640	.749	.634
SMO	.760	.664	.759	.710	**.748**	.696	.746	.654	**.755**	.645	.746	**.643**	.745	.645
SPegasos	.764	.674	**.761**	.703	.747	.685	**.749**	.647	**.755**	.647	.749	**.643**	.747	.647
VotedPerceptron	.762	.682	.750	.703	.740	.700	.730	.653	.732	**.651**	.730	.635	.721	**.650**
Logistic	.753	**.690**	.751	**.722**	.736	**.706**	.725	**.665**	.730	.650	.728	.642	.728	**.650**
SimpleLogistic	.758	.674	.751	.714	.738	.676	.747	.644	.750	.645	.752	.639	.749	.632
LMT	**.765**	.684	.760	.694	.744	.685	.748	.638	.754	.642	.752	.634	**.751**	.637
RandomForest	**.765**	.684	**.761**	.698	.742	.687	**.749**	.634	.754	.636	**.753**	.627	.746	.637

Bolded results indicate the best performance achieved for each threshold value.

**Table 4:** Confusion Matrices for 10-fold cross-validation experiments.

	*th *> 49		*th *> 59		*th *> 74		*th *> 84		*th *> 89		*th *> 94		*th *> 99		
	
	predicted classes														true classes
	
	I	NI	I	NI	I	NI	I	NI	I	NI	I	NI	I	NI	
NaïveBayes	984	489	809	355	672	306	426	248	410	235	394	223	392	220	I
	324	1149	267	897	234	744	153	521	150	495	143	474	142	470	NI

J4.8	989	484	824	340	674	304	430	244	414	231	393	224	394	218	I
	319	1154	270	894	230	748	*141*	533	*135*	510	131	486	131	481	NI

SMO	980	493	812	352	679	299	434	240	417	228	**397**	220	395	217	I
	313	1160	*261*	903	228	750	145	529	*135*	510	133	484	133	479	NI

SPegasos	981	492	812	352	679	299	435	239	417	228	**397**	220	395	217	I
	313	1160	*261*	903	228	750	146	528	137	508	133	484	131	481	NI

VotedPerceptron	998	475	828	336	677	301	432	242	**421**	224	396	221	**402**	210	I
	324	1149	279	885	231	747	154	520	155	490	144	473	152	460	NI

Logistic	**1005**	468	**832**	332	**687**	291	**440**	234	419	226	396	221	399	213	I
	334	1139	279	885	250	728	164	510	156	489	146	471	145	467	NI

SimpleLogistic	987	486	814	350	669	309	428	246	414	231	392	225	388	224	I
	311	1162	271	893	*220*	758	149	525	*135*	510	129	488	130	482	NI

LMT	119	45	93	37	75	34	428	246	414	231	390	227	389	223	I
	1700	5673	1882	6143	2032	6386	150	524	135	510	129	488	130	482	NI

RandomForest	985	488	817	347	682	296	427	247	410	235	388	229	386	226	I
	*309*	1164	264	900	233	745	142	532	137	508	*128*	489	*126*	486	NI

*I *indicates the positive class, i.e. instances reporting ILI cases, while *NI *indicates the negative class, i.e. non-ILI instances. Bolded results indicate the highest true positive (TP) value obtained for each threshold value; results highlighted in italics indicate the lowest false positive (FP) value obtained for each threshold value.

Figure 3 suggests that overall, all classifiers achieve better performance when a loose definition of influenza related tweets is used, i.e. when 49 ≤ *th *≤ 74, with the best F-measure value achieved by multinomial logistic regression classifier (Logistic, 0.736 F-measure at *th *= 59). When stricter threshold values are used, then the F-measures of all classifiers decrease, this decrement occurring somewhere in the interval of threshold values between 74 and 84, with F-measures being overall stable between 84 and 99. The values of precision and recall (Table 3) report a similar finding, although losses in performance are different across precision and recall for different classifiers. For example, the Random Forests classifier exhibits a higher loss in precision than that in recall when passing from a threshold value of 49 to 99. Conversely, the Naive Bayes classifiers exhibits similar losses in performance across both precision and recall when considering threshold values of 49 and 99.

Figure 3 confirms findings of previous studies, that Support Vector Machine approaches are generally better than Naive Bayes in determining if a tweet is reporting ILI cases, e.g. [8]. However, our study reports on the performance of a wider range of classifiers. The empirical results show that there are a number of classifiers that guarantee performance that are usually bounded by those of SVMs and Naive Bayes, and exceed SVMs performance in specific circumstances. For example, while the multinomial logistic regression classifier (Logistic) generally achieves F-measures higher than Naive Bayes but lower than SVMs, it does improve over SVMs when the threshold value is 59. The multinomial logistic regression classifier in fact proves to be comparable to the best SVM approach (SMO - polynomial kernel and sequential minimal optimisation algorithm) when a relaxed definition of influenza is used to classify tweets. When a more strict definition of flu-related tweets is adopted, the performance of Logistic degrades, indicating poor robustness across threshold values of this logistic regression classifier for this task. A similar conclusion can be drawn for the other logistic regression approaches investigated in this study. In fact, the linear logistic regression classifier (denoted SimpleLogistic) produces F-measures comparable to that of SVMs when the threshold is set to 59, 89, 94; however it does perform poorly with other threshold values, i.e. 49, 74, 99.

We now examine the results reported in Table 4 i.e. the confusion matrices produced by each classifier in the cross-validation experiments. Confusion matrices provide a crude but finer-grain understanding of classifier performance than summary measures like F-measure. For each matrix in the table, the first row indicates the number of tweets that are influenza-related according to the ground truth annotations and their classification value according to the studied classifiers (left column: classified as influenza, i.e. true positive (TP) cases; right column: classified as non-influenza, i.e. false negative (FN) cases). Vice versa, the second row indicates the number of tweets that have been assessed as not reporting influenza cases; the leftmost value corresponds to non-influenza tweets that have been erroneously classified as being influenza-related false positive - FP), while the rightmost value corresponds to non-influenza tweets that have been correctly classified (i.e. true negative - TN).

If identifying more influenza-related tweets is of key importance, then the best classifier that achieves this is the one with the larger number of TP instances (or vice-versa, the lower number of FN). In Table 4 the highest TP value has been highlighted in bold for each threshold value. For low-mid threshold values (49 ≤ *th *≤ 84), the multinomial logistic regression classifier (Logistic) returns the highest number of TP instances. For higher threshold values, the highest number of TP instances is returned by the voted perceptron (*th *= 89, 99) and SVMs classifiers (*th *= 94), although Logistic provides a very similar result.

If on the other hand producing a low amount of false positive influenza alerts is of key importance, then the most suitable classifier is the one that produces the lowest amount of FP instances (or vice-versa, the highest number of TN); this has been highlighted in italics in Table 4. While the Random Forests classifier produces the lowest number of FP instances at low and high threshold values (*th *= 49, 94, 99), not one classifier exhibits consistently lower FP instances for mid values of the threshold (59 ≤ *th *≤ 89).

#### Effectiveness on unseen data

The results obtained by the classifiers when validated against unseen data are analysed next. Tables 5 and 6 report respectively the F-measure values and the confusion matrices produced by the classifiers.

**Table 5:** F-measure values with respect to the threshold level obtained by the studied classifiers when evaluated on unseen data.

	*th *> 49	*th *> 59	*th *> 74	*th *> 84	*th *> 89	*th *> 94	*th *> 99
NaiveBayes	.110	.084	.064	.036	.034	.031	.031
J4.8	.119	.083	.064	.037	.035	.033	.033
SMO	.118	.086	.067	.038	.035	.033	.033
SPegasos	.050	.101	.058	.108	.073	.079	.079
VotedPerceptron	.117	.085	.066	.037	.034	.032	.031
Logistic	.117	.085	.066	.037	.033	.030	.030
SimpleLogistic	.120	.088	.068	.037	.035	.033	.033
LMT	.120	.089	.068	.037	.035	.033	.033
RandomForest	.119	.089	.066	.038	.035	.032	.033

**Table 6:** Confusion Matrices obtained when testing on unseen data.

	*th *> 49		*th *> 59		*th *> 74		*th *> 84		*th *> 89		*th *> 94		*th *> 99		
	
	predicted classes														true classes
	
	I	NI	I	NI	I	NI	I	NI	I	NI	I	NI	I	NI	
NaiveBayes	110	54	91	39	74	35	44	31	43	29	39	30	39	29	I
	1725	5648	1950	6075	2124	6294	2298	6762	2414	6707	2413	6767	2416	6775	NI

J4.8	118	46	**95**	35	77	32	45	30	43	29	40	29	40	28	I
	1701	5672	2054	5971	2219	6199	2300	6760	2324	6797	2351	6829	2353	6838	NI

SMO	117	47	94	36	**78**	31	**46**	29	43	29	40	29	40	28	I
	1696	5677	1954	6071	2133	6285	2320	6740	2324	6797	2351	6829	2353	6838	NI

SPegasos	5	159	10	120	5	104	7	68	4	68	5	64	5	63	I
	*30*	7343	*58*	7967	*57*	8361	*48*	9012	*33*	9088	*53*	9127	*53*	9138	NI

VotedPerceptron	**121**	43	**95**	35	73	36	**46**	29	**44**	28	**42**	27	**41**	27	I
	1788	5585	2000	6025	2025	6393	2382	6678	2465	6656	2497	6683	2514	6677	NI

Logistic	**121**	43	**95**	35	**78**	31	**46**	29	43	29	38	31	40	28	I
	1776	5597	2002	6023	2170	6248	2392	6668	2463	6658	2396	6784	2529	6662	NI

SimpleLogistic	119	45	89	41	76	33	45	30	43	29	40	29	40	28	I
	1700	5673	1855	6170	2060	6358	2189	6871	2324	6797	2351	6829	2353	6838	NI

LMT	119	45	95	35	76	33	45	30	43	29	39	30	40	28	I
	1700	5673	1912	6113	2045	6373	2300	6760	2324	6797	2263	6917	2353	6838	NI

RandomForest	119	45	94	36	77	32	44	31	41	31	38	31	39	29	I
	1725	5648	1881	6144	2153	6265	2187	6873	2242	6879	2263	6917	2271	6920	NI

*I *indicates the positive class, i.e. instances reporting ILI cases, while *NI *indicates the negative class, i.e. non-ILI instances. Bolded results indicate the highest true positive (TP) value obtained for each threshold value; results highlighted in italics indicate the lowest false positive (FP) value obtained for each threshold value.

The classifiers exhibit lower F-measures when validated on the unseen dataset than when tested in the cross-validation settings. This is because the dataset used for cross-validation was balanced across the two classes (same number of influenza-related and non influenza-related instances), while the dataset used in this second experiment is heavily imbalanced towards the negative class. This means that there are many more non-influenza tweets than the influenza ones: in fact, the percentage of influenza-related tweets in this dataset varies across the different threshold values and ranges between 2.22% for *th *= 49 and 0.74% for *th *= 99 (while in the balanced dataset was 50%). Nevertheless, the results confirm the observation made in the cross-validation settings that automatically classifying tweets under a loose definition of influenza is easier than under the strict settings, i.e. all classifiers obtain higher F-measures for low threshold values than for high threshold values. The SPegasos variation of SVM does however constitute an exception, as inconsistent F-measure values are measured across the range of threshold values; in particular, performance yielded at the lowest threshold are worse than that at any other threshold value. The values reported in the confusion matrices for SPegasos (Table 6) highlight that this classifier is unable to correctly identify a large percentage of positive instances (TP) while it correctly identifies non-influenza cases (TN) at a higher rate than other classifiers, therefore yielding often larger values of F-measure due to the imbalanced nature of the dataset.

The results discussed in the previous paragraph suggest that considering F-measure values may lead to performance underestimation: an error rate for negative instances has a proportional larger contribution than a similar error rate on positive instances. To avoid this, we calculate the balanced accuracy yielded by each classifier under the different threshold settings. Balanced accuracy *Â *(i.e. the average accuracy obtained on either class) is defined as [24]:

(1) $$Â=\frac{0.5*TP}{TP+FN}+\frac{0.5*TN}{TN+FP}$$

When contrasted with the standard accuracy measure, balanced accuracy presents the advantage that *Â *is high if a classifier performs equally well on both classes, while *Â *is low when high (standard) accuracy is obtained only because the classifier is advantaged or penalised by an imbalanced dataset, like in this case. A majority class classifier (in this case a classifier that assigns every instance to the negative class) and a minority class classifier (all instances assigned to the positive class) will obtain a balanced accuracy equivalent to chance (i.e. 0.5). The balance accuracy obtained by the classifiers investigated in this study is reported in Table 7.

**Table 7:** Balanced accuracy values (*Â*) with respect to the threshold level obtained by the studied classifiers when evaluated on unseen data.

	*th *> 49	*th *> 59	*th *> 74	*th *> 84	*th *> 89	*th *> 94	*th *> 99
NaiveBayes	.718	.729	.713	.667	.666	.651	.655
J4.8	.744	.737	.721	.673	**.671**	.662	**.666**
SMO	.742	.740	**.731**	**.679**	**.671**	.662	**.666**
SPegasos	.513	.535	.520	.544	.526	.533	.534
VotedPerceptron	**.748**	.741	.715	.675	.670	**.668**	.665
Logistic	**.748**	.741	.729	.675	.664	.645	.657
SimpleLogistic	**.748**	.727	.726	**.679**	**.671**	.662	**.666**
LMT	**.748**	**.746**	.727	.673	**.671**	.659	**.666**
RandomForest	.746	.744	.725	.673	.662	.652	.663

Values of balanced accuracy generally decrease as the threshold values increase: this confirms the previous analysis. In addition, balance accuracy reveals that the SVM instance implemented by SPegasos performs just above chance across all threshold values. This suggests that the model learnt by SPegasos on the cross-validation data is poorly applicable to the unseen data contained in the second dataset. Performance of other classifiers do however scale on unseen data. The best values of balance accuracy across each threshold value are highlighted in bold in Table 7. The observation that the Naive Bayes classifiers constitutes a lower bound in classification performance in the cross-validation experiments is confirmed in this second experimental setting. The SMO implementation of SVM classifier is confirmed to provide consistently high performance. The finding observed under the cross-validation settings that the multinomial regression classifier Logistic performs similar to SMO for low threshold value while losing effectiveness for higher vales is confirmed in this experiment. Vice versa, in this second experiment the other linear regression classifier, SimpleLogistic, is found to provide similar results to SMO across the different threshold values.

## Conclusions

In this paper we have investigated the performance of machine learning classifiers for the task of detecting Twitter messages that mention possible cases of influenza or ILI. Our experiments considered a number of standard textual features, such as word tokens, stems and n-grams; in addition, we did consider features that are specific to Twitter messages, such as the presence of Twitter usernames, hashtags (i.e. #String), URLs and emoticons. The creation and investigation of new alternative features is left for future research.

Previous studies have shown the effectiveness of SVMs over Naive Bayes classifiers. While our study confirms this result, we show that Naive Bayes's performance can often be considered as the lower bound of a wide range of alternative classifiers and that there are a number of classifiers that perform similarly (or better under specific settings) than SVMs. In particular, the instance of SVM with linear kernel and stochastic gradient descent (SPegasos) tested in our study showed limited robustness when tested on a heavy imbalanced unseen dataset, although confirming good performance on cross-validation experiments with balanced data.

Differences in performance between the cross-validation experiments and those on unseen data may highlight the importance of the training methodology used to form the classifiers, and in particular whether to balance or stratify the datasets used during the training and testing phases. Chai et al [25] found that classification methods trained, validated, and tested on balanced datasets overestimated classification performance when compared with testing on imbalanced (stratified) data. Similar results were found in our study, where classifiers' F-measure in the cross-validation experiments (with a balanced dataset) were higher than those achieved in the unseen dataset experiments (with an imbalanced dataset). To overcome this issue and present a meaningful analysis of the result obtained on unseen data, we used the balanced accuracy measure [24], that overcomes the issue of a biased classifier that has taken advantage of an imbalanced test set, like in the case for the SPegasos classifier in our experiments. We leave further investigation and analysis of training/testing methodology designs to future work.

Finally, the results also reveal that often there is only limited difference in performance across the different investigated classifiers. This suggests that future research efforts for the detection of ILI related tweets should be directed beyond improving machine learning techniques, in particular addressing how disease outbreak monitoring systems should cope with false positive notifications produced by the proposed automatic methods. Knowing the exact number of true ILI-related tweets may be not necessary in the settings of a disease outbreak monitoring system, as increases or decreases in trends of tweets classified as likely to be ILI-related may be sufficient to correctly suggest disease outbreaks. This hypothesis requires further investigation and is left for future work.

## Competing interests

The authors declare that they have no competing interests.

## Authors' contributions

GZ, SK, AN and JB conceived the study and participated in its design and coordination of the research. GZ, SK, AN, JB and MH contributed to the design of the Twitter annotation tool, Twitter annotations, and the interpretation of the results. GZ and MH developed, modelled, and performed evaluations and statistical analysis. MC provided the Twitter data set generated by the ESA-AWTM software. All authors have contributed to the drafting of the manuscript and have read and approved the final version.

## Declarations

The costs of publication associated to this article were funded by the Australian e-Health Research Centre.

This article has been published as part of *Health Information Science and Systems *Volume 3 Supplement 1, 2015: Proceedings of the Health Informatics Society of Australia Big Data Conference (HISA 2013). The full contents of the supplement are available online at http://www.hissjournal.com/supplements/3/S1/
